# Post-Translational Modification and Secretion of Azelaic Acid Induced 1 (AZI1), a Hybrid Proline-Rich Protein from Arabidopsis

**DOI:** 10.3390/ijms17010085

**Published:** 2016-01-12

**Authors:** Andrea Pitzschke, Hui Xue, Helene Persak, Sneha Datta, Georg J. Seifert

**Affiliations:** 1 Department of Cell Biology, Division of Plant Physiology, University of Salzburg, Hellbrunner Strasse 34, Salzburg A-5020, Austria; 2 Department of Applied Genetics and Cell Biology, University of Natural Resources and Life Sciences, Muthgasse 18, Vienna A-1190, Austria; hui.xue@boku.ac.at (H.X.); Helene.Persak@agrana.com (H.P.); sneha_datta@yahoo.co.in (S.D.)

**Keywords:** hybrid proline-rich proteins (HyPRPs), glycosylation, AZI1, post-translational modification, arabino galactan proteins (AGPs)

## Abstract

*Arabidopsis* EARLI-type hybrid proline-rich proteins (HyPRPs) consist of a putative N-terminal secretion signal, a proline-rich domain (PRD), and a characteristic eight-cysteine-motif (8-CM). They have been implicated in biotic and abiotic stress responses. AZI1 is required for systemic acquired resistance and it has recently been identified as a target of the stress-induced mitogen-activated protein kinase MPK3. AZI1 gel migration properties strongly indicate AZI1 to undergo major post-translational modifications. These occur in a stress-independent manner and are unrelated to phosphorylation by MAPKs. As revealed by transient expression of AZI1 in *Nicotiana benthamiana* and *Tropaeolum majus*, the *Arabidopsis* protein is similarly modified in heterologous plant species. Proline-rich regions, resembling arabinogalactan proteins point to a possible proline hydroxylation and subsequent *O*-glycosylation of AZI1. Consistently, inhibition of prolyl hydroxylase reduces its apparent protein size. AZI1 secretion was examined using *Arabidopsis* protoplasts and seedling exudates. Employing Agrobacterium-mediated leaf infiltration of *N. benthamiana*, we attempted to assess long-distance movement of AZI1. In summary, the data point to AZI1 being a partially secreted protein and a likely new member of the group of hydroxyproline-rich glycoproteins. Its dual location suggests AZI1 to exert both intra- and extracellular functions.

## 1. Introduction

Plant hybrid proline-rich proteins (HyPRPs) are characterized by an N-terminal secretion signal peptide, a proline-rich domain (PRD) and a conserved eight-cysteine motif (8-CM) at the C-terminus. This latter hydrophobic C-terminal domain is also found in lipid transfer proteins (LTPs), amylase inhibitors and 2S albumins [1,2]. Some tertiary structure data exists for 8-CM-containing proteins, though not for HyPRPs. However, a similar folding of the 8-CM region may be assumed [1]. Functional information cannot yet be deduced, and the 8-CM may merely act as a scaffold carrying specific functional elements specific to the individual subgroups of the 8 CM family [2]. Thus, though the HyPRP sequence features are well-defined, little knowledge exists on protein functions and localization. The C-terminal 8-CM is related to so-called non-specific lipid transfer proteins (ns-LTPs). Classical LTPs do not have a PRD [3]. HyPRPs are encoded by 29 genes in *Arabidopsis*, and 31 genes in rice [3].

One subfamily of *Arabidopsis* *thaliana* HyPRPs is called the EARLI1-type subfamily. The name derives from one member, EARLI1 (At4g12480). EARLIs, along with several other, unrelated genes, named *Early Arabidopsis Aluminium Induced* genes, had initially been discovered in a cDNA screen study on seedling responses to aluminium in 1998 [4]. Based on sequence homology, HyPRPs related to EARLI1 could subsequently be identified. Four of them are particularly closely related and form a separate branch in the HyPRP phylogenetic tree [2,5], (for details on phylogenetic relations to HyPRPs, also from other plant species, see for e.g., [1]). From refined sequence alignments it became apparent that the major distinguishing feature among the four EARLI1-type HyPRPs is the number of repetitive motifs in their proline-rich domain [5,6].

The four EARLI1-type genes are clustered in a tandem array on chromosome 4 (At4g12470-At4g12500). Differences exist among EARLI1-type gene expression profiles, suggesting that despite high homology at the protein level, full functional redundancy is unlikely. Three members (At4g12470, At4g12480 and At4g12500) have been implicated in the control of flowering time and lignin biosynthesis [7]. EARLI1 (At4g12480) assists germination and seedling development under cold and salt stress [8]. One representative, At4g12470, is also known as AZI1 (*azelaic acid induced gene 1*), called after its unique response to the systemically active compound azelaic acid [9]. Furthermore, *AZI1* is specifically downregulated in response to combinatorial treatment with abiotic and biotic stresses [10]. AZI1 deficiency correlates with diverse physiological defects: Mutants are compromised in abiotic and biotic stress responses (see below). It has recently been shown that AZI1 interacts with the LTP family member DIR1 (*defective in induced resistance 1*) *in planta* [11].

In line with Northern blot analyses revealing activation of *EARLI1*-type genes by low temperature, the respective proteins were found to accumulate in cold-exposed plants. These observations derive from immunoblot analyses conducted with an anti-EARLI1-specific antibody, which recognized multiple immunoreactive protein bands migrating at substantially larger than the expected size [5]. Furthermore, these “higher order complexes” were found to be persistent even under strongly reducing conditions [5]. Until now, the chemical moieties or modifications accounting for the unusual gel migration properties of EARLI-type HyPRPs are elusive.

Mitogen-activated protein kinases (MAPKs) mediate numerous cellular processes via phosphorylation of their target proteins. In particular, *Arabidopsis* MPK3 activity is induced by various abiotic and biotic stresses, such as wounding, osmotic stress and microbial elicitor treatment [12,13,14,15]. The majority of known MPK3 substrates are transcription factors, and MPK3 modulates target protein properties in a number of ways [16]. We recently reported on the EARLI-type HyPRP AZI1 as a MPK3 target, and we proposed a role of MPK3 as a positive regulator of AZI1 abundance [6,17]. AZI1 overexpression enhances salt stress tolerance [6]. Besides being hypersensitive to salt stress [6], *azi1* mutants are compromised in systemic acquired resistance [9], for which also MPK3 is indispensable [18].

In immunoblot experiments with anti-myc antibody, C-terminally tagged AZI1-myc protein produced in stable transgenic *Arabidopsis* plants was found to migrate as a 36/38 kD double band. The fusion protein thus exhibits a substantial difference between apparent and calculated size (29.4/31.9 kD; secretion peptide cleaved/noncleaved). Theoretically, this could be attributable to intramolecular cysteine linkages or homodimerisation of AZI1-myc molecules via cysteine residues of the 8-CM motif. However, retarded gel migration of AZI1-myc protein (detected by anti-myc) occurred irrespective of the presence or absence of high concentrations of reducing agents (DTT or β-mercapto ethanol) [6]. Because AZI1 contains a predicted N-terminal secretion signal, another explanation might be modification by *N*-linked glycans. Again, this is unlikely because AZI1 is devoid of *N*-glycosylation motifs (Asn-X-Ser/Thr).

The largest group of structural cell wall proteins are known as hydroxyproline-rich glycoproteins (HRGPs) [19] that were further classified into moderately *O*-glycosylated extensins, highly *O*-glycosylated arabinogalactan proteins (AGPs) and lightly *O*-glycosylated proline-rich proteins (PRPs, not to be confused with HyPRPs). Extensin proteins often contain Ser-Hyp_4_-repeats where each Hyp residue is glycosylated with one to four arabinosyl residues and individual galactosylated serines [20]. AGPs, on the other hand frequently contain stretches of isolated Hyp residues separated by Ser, Thr, Ala, or Gly [21], however more complex motifs have been reported [22,23]. Based on numerous HRGP peptide sequences, the Hyp contiguity hypothesis states that continuous Hyp residues are (oligo)-arabinosylated, and discontiguous Hyp-residues are galactosylated with type II arabinogalactans [24]. Type II arabinogalactan (AG II) contains a backbone of α-(1-3) linked galactose with α-(1-6) galactosyl side chains or -kinks [25,26]. This basic structure is further substituted with arabinosyl and various other glycosyl residues and may display a large variation in molecular weight. Because no upper molecular weight limit of AG II is known, a single AG II modification might lead to a substantial increase in molecular weight. One member of the *Arabidopsis* AGP family whose glycosylation structure is known in detail, AGP31 [27], displays high homology to AZI1 [6]. Apart from phosphorylation, posttranslational modifications in AZI1 are elusive. Several Ser-Pro motifs in its Pro-rich N-terminus suggest that AZI1 might be hydroxylated and in turn be AG II modified.

Here, we studied post-translational modification(s) accounting for AZI1 gel retardation. We assessed whether these modifications occurred in a MAPK-, stress treatment- and/or plant species-dependent manner. Given the high similarity of AZI1 to some AGPs and PRPs, *O*-glycosylation was assessed in particular. We furthermore investigated AZI1 secretion using a protoplast-based system as well as *Arabidopsis* seedling exudates. Employing *Agrobacterium*-mediated leaf infiltration of *N. benthamiana* we attempted to gain insight into long-distance movement of AZI1.

## 2. Results

### 2.1. AZI1 Retarded Mobility in Protein Gels

#### 2.1.1. AZI1 Gel Migration Is Unrelated to MAPK Phosphorylation

At least two explanations for the major difference between calculated and apparent protein sizes seem plausible: (i) Intramolecular disulfide bridges in EARLI1-type proteins are particularly resistant to reducing conditions; (ii) The proteins undergo redox-independent posttranslational modification(s). One suspect candidate involved in these modifications would be MPK3. MPK3 is known to regulate AZI1 levels, to interact with AZI1 *in vivo*, and to phosphorylate AZI1 *in vitro*. Despite these connections, MPK3 and MPK3-mediated phosphorylation can be excluded as determinant or trigger of AZI1 gel retardation. This conclusion derives from the fact that AZI1-myc proteins produced in Col-0 or *mpk3* mutants show the same profile [6]. However, MPK3 may not be the only MAPK able to phosphorylate AZI1. In fact, also MPK6, the closest homolog of MPK3, interacts with AZI1 *in vitro* [6]. The two kinases are known to have largely, though not fully, overlapping functions [14]. Unfortunately, *mpk3/mpk6* mutants are embryo-lethal [28], thus hampering AZI1 analysis in a MPK3 *and* MPK6-deficient background. One strategy which in part circumvents this limitation is to specifically block substrate phosphorylation.

**Figure 1:**
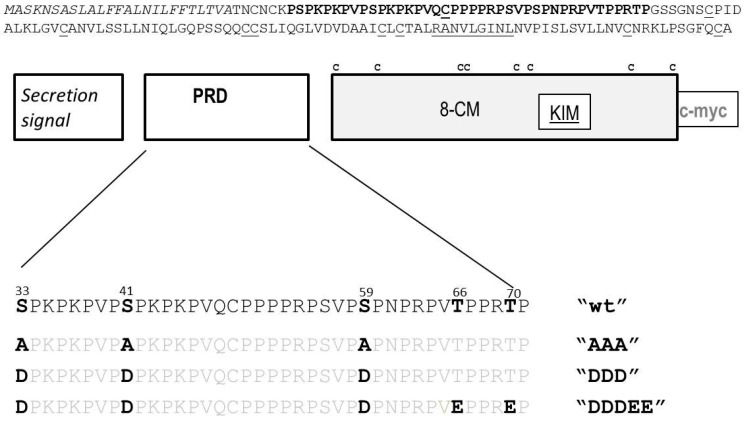
AZI1 primary protein structure. (**Top**): The protein sequence of AZI1 is shown with putative secretion signal in italics, proline rich domain (PRD) in bold and cysteine residues (C) and putative MAP kinase interaction motif (KIM) underlined; (**Middle**): A cartoon of the protein motifs, with position of cysteine residues within the 8-cysteine-motif (8-CM) indicated); (**Bottom**): The PRD is shown in detail, with all five putative MAPK phosphorylation sites (serine, S; threonine, T) highlighted. Dephosphomimetic (AAA) and phosphomimetic AZI1 variants (DDD, DDDEE), carrying amino acid substitutions to alanine (A), aspartate (D) or glutamate (E) are displayed below the AZI1 wild type (wt) sequence. All constructs used carry a C-terminal c-myc tag.

**Figure 2:**
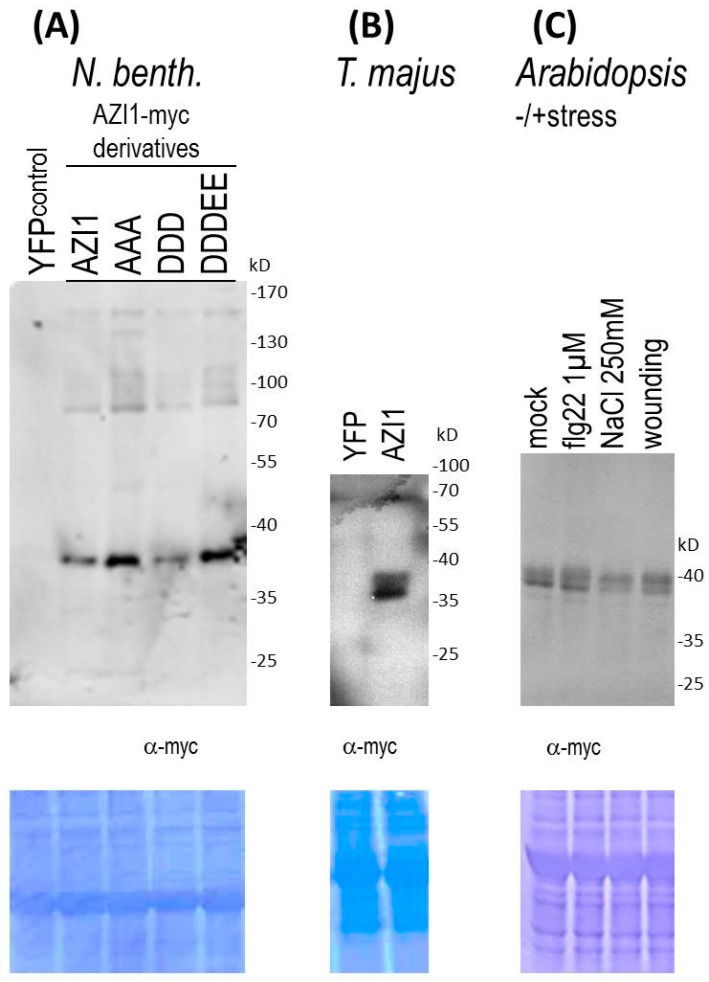
AZI1 gel retardation is MAPK-, plant species- and stress-independent. Transient expression of AZI1-myc in *N. benthamiana* (**A**) and *T. majus* (**B**). Leaves were infiltrated with agrobacteria carrying the respective constructs. Proteins were extracted five days after infiltration, separated by SDS-PAGE and detected by immunoblot analysis with anti-myc antibody. Differences in signal intensity between AZI1 derivatives (panel **A**) likely result from position effects/transformation efficiency, not from altered protein stability; (**C**) Arabidopsis seedlings stably expressing the AZI1-myc transgene were exposed to stress prior to immunoblot analysis. **Upper** panels: Anti-myc detection; **lower** panels: Membranes after subsequent staining with Coomassie brilliant blue for documentation of protein loading.

Although in general a protein’s molecular weight increases only slightly upon replacements of free hydroxyl to phosphate groups, phosphorylation may trigger subsequent additional major post-translational modifications. Employing a mutagenesis approach we therefore tested whether the protein’s retarded gel migration was related to phosphorylation by *any* MAPK. AZI1 contains one kinase interaction motif (KIM), located in the 8-CM domain and five sites matching the minimal consensus for MAPK phosphorylation, *i.e.*, Ser-Pro or Thr-Pro dipeptides, located in the PRD [16]. The five putative phosphorylation sites (Ser33, Ser41 Ser59, Thr66 and Thr70), were therefore replaced, and the resultant constructs (Figure 1) used for transient ectopic expression in *N. benthamiana* leaves. SDS-PAGE and anti-myc immunoblot analyses documented successful expression of AZI1-myc and its derivative (Figure 2A). Similar patterns of myc-specific immunoreactive signals—a dominant 38 kD- and a minor 36 kD-sized band—were found in all AZI1-related samples, but not in the control (YFP). These data imply that AZI1 gel migration properties are neither directly nor indirectly related to phosphorylation by MAPKs. In the AZI1 primary protein sequence numerous additional serine (15) and threonine (4) residues exist, which may represent potential phosphorylation sites for other types of kinases. A possible impact of modifications at these 19 residues on AZI1 gel migration characteristics remains to be assessed in future work.

#### 2.1.2. Substitutions at MAPK Target Sites Modify AZI1 Function

As outlined above, replacement of putative MAPK target sites did not notably change AZI1 protein migration. However, phosphorylation might yet be relevant for AZI protein function. A useful parameter for assessing this is the known AZI1 overexpression phenotype, *i.e.*, enhanced salt stress tolerance. We therefore selected two *AZI1* variant transgenes, *AZI1-AAA* and *AZI1-DDDEE*, for constitutive overexpression in *Arabidopsis*. Stable transgenic lines exhibiting similar expression levels as the native AZI1-myc line were selected to compare germination performances under high-salinity stress (Figure S1). Under non-stress conditions, germination in all AZI1 transgenics (AZI1 wt, -AAA, -DDDEE) was similar to that of control Col-0 seeds. Corroborating previous findings, germination was reduced and delayed in *azi1* mutants. On salt medium (150 mM NaCl), overexpression of AZI1 in its native form (AZI1 wt) improved germination. AZI1-DDDEE lines also performed better as compared to control Col-0 plants, but stayed behind AZI1 wt lines. Contrasting the germination-enhancement seen in AZI1 wt and AZI1-DDD lines, salt hypersensitivity was observed in dephosphomimetic AZI1-AAA overexpressors. In summary, these observations re-confirm the role of AZI1 in salinity stress adaptation and suggest phosphorylation to be relevant for AZI1 function. The phenotype arising from AZI1-AAA overexpression is indicative of a dominant negative effect. It resembles the situation described in a previous study on (de-)phosphomimetics of another MPK3 substrate, transcription factor MYB44 [29]. A possible explanation is that the dephosphomimetic variant is non-functional but competes with endogenous AZI1 for binding sites in proteins or other molecules critical in salt stress responses. Alternatively, AZI1-AAA forms dimers with and thus inactivates endogenous AZI1. These are plausible explanations, but will need to be tested by complementation studies involving overexpression of AZI1 and its variants in the salt-sensitive *azi1* mutant.

#### 2.1.3. Indications of AZI1-Modifying Machineries in Heterologous Plant Species

Unlike the situation in *Arabidopsis* and *N. benthamiana*, AZI1 recombinant proteins produced in *E. coli* migrate at the size expected for the unmodified protein [6], indicating that eukaryotic/*in planta* modification is required for the observed molecular weight. We asked whether AZI1 was similarly modified also in other plants, using a recently established transgenesis system [30]. AZI1-myc was transiently expressed in *Tropaeolum majus*, a member of the order Brassicales. Protein extracts from transformed *T. majus* leaves exhibited the characteristic 36/38 kD band (Figure 2B). The data from *N. benthamiana* and *T. majus* suggest that AZI1 from *Arabidopsis* undergoes the respective post-translational modification also in heterologous plant systems. In other words, plants but not bacteria contain functional AZI1-modifying machineries.

#### 2.1.4. AZI1 Gel Retardation Is Stress-Independent

Given the known roles of AZI1 in stress tolerance, we asked whether the apparent AZI1 protein size would vary in a stress-dependent manner. Three scenarios can be imagined. (i) The protein’s apparent size remains constant; (ii) AZI1 may engage in even larger SDS/DTT-persistent complexes; (iii) the modification seen in the non-stressed state may be cleaved thus enhancing the protein’s gel mobility. *Arabidopsis* seedlings overexpressing the AZI1-myc transgene were exposed to four different conditions for one day: standard medium (mock control), saline stress (NaCl 250 mM), wounding, and biotic stress (bacterial elicitor treatment, flg22 1 µM). Subsequent analysis of protein extracts from these four samples revealed the characteristic 36/38 kD size profile, irrespective of the type of plant treatment. (Figure 2C). Seedlings exposed to shorter stress periods (few minutes, several hours) also showed this pattern. Similarly, AZI1 apparent protein size remained constant throughout various developmental stages and tissues (not shown). In summary, the modification(s) accounting for retarded AZI1 gel migration appear to occur in a stimulus-, age- and organ-independent manner.

### 2.2. O-Glycosylation Studies

#### 2.2.1. Glycosylation—Theoretical Considerations

We consider the presence of covalently bound sugar chains in AZI1 a very plausible explanation for the protein’s size enigma. Though not providing final evidence, there are several protein features in favor of this AZI1 glycosylation hypothesis: First, AZI1 displays eukaryotic-specific retarded gel migration [6]; Second, presence of a secretion signal as well as microscopic detection in endoplasmic reticulum [11] and plasma membrane [5,6] suggest AZI1 to enter the biosynthetic-secretory pathway via ER and Golgi, which are the main loci of protein glycosylation; Third, AZI1 has sequence similarity with known glycoproteins. Since AZI1 lacks Asn-X-Ser/Thr motifs known to be targeted by *N*-glycosyltransferases [31], only *O*-linked glycosylation is further considered. Although different types of *O*-glycans have been recently observed in various kingdoms [32], the predominant mode involves linkage of L-arabinose or d-galactose to hydroxyproline, giving rise to extensin-like short arabinosides and large branched type II arabinogalactans, respectively.

**Figure 3:**
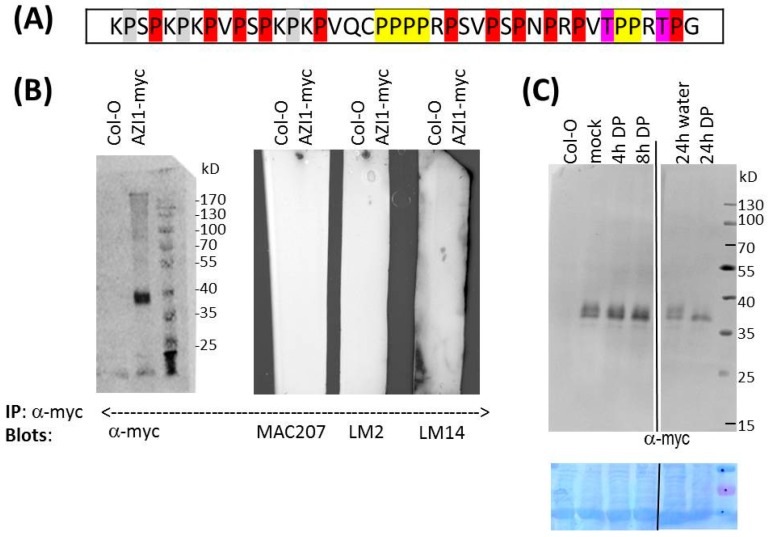
AZI1 glycosylation—theoretical considerations and experimental approaches. (**A**) Potential glycomodules in the AZI1 Pro-rich region (residue 31 to 72) according to the Hyp contiguity hypothesis [32] and the assumption of general Pro-hydroxylation, unless excluded by Rule 1; Rule 1: Lys-Pro, Leu-Pro, Tyr-Pro are never hydroxylated (highlighted in grey) but Pro-Val is always hydroxylated (in case of conflict we decided to prioritize the latter). Rule 2: Contiguous Hyp is arabinosylated (highighted yellow). Rule 3: discontiguous Hyp is AG II modified (highlighted red). Under the applied rules, T66E or T70E substitutions (highlighted in purple, see Figure 1) permits prolyl hydroxylation and sugar attachment. In summary, the assumed modifications are: arabinosylation at six positions (yellow) and galactosylation at up to eleven positions (red). The glycan size cannot be predicted and for each AG glycan might range in size from single galactosyl residues to high molecular weight AG II polysaccharides [27]. AZI1 molecules carrying some of the theoretically possible modifications would easily exceed the predicted peptide mass; (**B**) Glycan-directed antibodies do not recognise AZI1-myc purified from plants. Protein extracts from 3-week-old Arabidopsis Col-0 wild type or AZI1-myc overexpressing plants were immunoprecipitated with mouse anti-myc antibody and proteinA-coupled magnetic beads. Captured proteins were analysed by SDS-PAGE and immunoblot detection using the indicated antibodies from rabbit. AZI1-myc can be successfully immunoprecipitated (**left**), but the protein is not recognised by anti-glycan-directed antibodies (**right**). MYC207, LM2 and LM14 did recognise positive control proteins (not shown); (**C**) Inhibition of prolyl hydroxylation alters AZI1 gel migration. Two independent experiments are shown. **Left** panel: 14-day-old *Arabidopsis* seedlings constitutively expressing a AZI1-myc transgene were incubated in standard medium supplemented with 100 µM 2,2-dipyridyl. Mock-treatment was for 4 h; **Right** panel: 10-day-old seedlings were incubated in tap water with or without 100 µM 2,2-dipyridyl for 24 h. Proteins were extracted, separated by SDS-PAGE and detected with anti-myc antibody. Protein loading was documented by staining of the PVDF membrane with Coomassie brilliant blue (**bottom**).

EARLI1-type proteins have a characteristic proline-rich domain whose pattern of proline residues is reminiscent of HRGPs (Figure 3A). We had noted some sequence homology between AZI1 and known glycoproteins [6]. However, the issue of glycosylation has not yet been addressed experimentally. Proline hydroxylation and subsequent modification with AG II glycans of AZI1 appears a realistic possibility.

#### 2.2.2. Is AZI1 an AGP?

The experimental diagnosis of *O*-glycosylation in plants is highly challenging. A general glyco-biochemical analytical approach is the enzymatic or chemical fragmentation of glycans or of glycoproteins followed by peptide sequencing or mass spectrometry (MS). In contrast to the specific removal of entire *N*-glycans by commercially available glycosyl-hydrolases [33], enzymatic determination of AG II glycans is a multi-step reaction that has previously only been successful for complex mg-scale AGP preparations [26,34]. Only highly abundant AGPs have been purified on a scale that permitted their mass-spectrometric analysis [21,27]. Several labs have recently assembled a panel of glycan-specific monoclonal antibodies (mAbs) that have proven invaluable for the analysis of complex cell wall polysaccharides and *O*-glycans. Although most epitopes are not precisely known, there is ample evidence for their cognate carbohydrate classes [35,36]. This is especially important for AGPs for which few alternatives of sensitive detection exist [37]. To gain insight into the hypothesized AZI1 *O*-glycosylation, proteins were extracted from control wild type and AZI1-myc overexpressing (stable, transgenic) *Arabidopsis* plants. AZI1-myc was subsequently immunoprecipitated (IP) using anti-myc antibody and proteinA-coupled magnetic beads. Immunoblot analysis of samples with anti-myc antibody documented successful isolation of AZI1-myc protein (Figure 3B, left). Similar to the crude extract, the purified protein migrated as 36/38 kD double band. However, no specific signal was obtained when testing the IP samples with antibodies (MAC207, LM2, LM14) directed against various AGP-related glycan epitopes [38,39] (Figure 3B, right). Similar immunoprecipitation/anti-glycan immunoblot analyses, using protein extracts from *N. benthamiana* transiently overexpressing AZI1-myc or AZI1-YFP, provided no indication for AZI1 to contain AGP-related carbohydrate epitopes (not shown). However, the absence of positive antibody reactions does not rule out the possibility of AZI1 glycosylation (see discussion).

#### 2.2.3. Pharmaceutical Approach: Inhibition of Proline Hydroxylation

*In vivo*, tunicamycin specifically blocks *N*-glycosylation [40], but no specific inhibitor for *O*-glycosylation is presently known. The indirect inhibition of *O*-glycosylation, however, is feasible: In AGPs, *O*-glycosylation occurs at hydroxyproline residues. The conversion of proline to hydroxyproline is catalyzed by prolyl hydroxylases. Enzyme activity can be blocked by 2,2-dipyridyl (DP), a chelator of the cofactor Fe^2+^ [41]. Consequently, AZI1 overexpressing plants were exposed to standard solution supplemented with 100 µM DP, and collected for protein analysis after 4 and 8 h. The mock-treated sample showed the expected 36/38 kD double band, while in DP-treated plants the 38 kD signal successively disappeared. Upon prolonged DP treatment (24 h) (Figure 3C), the 38 kD-band was not detectable any more, while signal intensity at 36 kD remained seemingly constant. Plants treated for 72 h still contained the single, 36 kD-sized band, and similar observations were made with AZI1/*mpk3* plants (not shown).

From these observations several conclusions can be drawn. (1) AZI1 carries one or several hydroxyproline residues; (2) Because hydroxylation adds merely 16 atomic mass units, even hydroxylation of all 24 possible proline residues would only marginally (<0.4 kD) increase the protein’s size. The observed changes upon DP-treatment therefore more likely result from inhibition of subsequent larger modifications, *i.e.*, *O*-glycosylation; (3) Because DP effects on AZI1/*mpk3* and AZI1/Col-0wt are similar, MPK3 is not involved in this modification; (4) The kinetics in the 38 kD band disappearance is a likely readout for AZI1 protein turnover. After eight hours the initially present protein has been fully degraded. The 36 kD band, being unaffected by DP treatment, may reflect the protein’s steady state level; (5) Proline hydroxylation does not affect AZI1 protein stability. As noted earlier, stability is largely controlled by MPK3 [6]; (6) AZI1’s apparent size (36 kD) is still substantially larger than the calculated size, even after long-term inhibition of prolyl hydroxylation. This is evidence for a multifaceted modification of the protein. AZI1 seemingly undergoes additional modifications, besides MAPK-mediated phosphorylation and *O*-glycosylaton at hydroxyproline residues.

### 2.3. Studies on AZI1 Secretion in Vivo

AZI1 has several properties that render it a candidate secretory protein: It carries an N-terminal secretion signal. AZI1-fluorescent protein fusions had been located in the ER (the site where secretion-destined proteins are processed) [11] and also at the cell periphery [5,6]. This prediction is also supported by proteomic studies that identify AZI1 in plasmodesmata and the plasma membrane [42,43]. Moreover, a role of AZI1 in the production or translocation of a mobile signal in the establishment of systemic acquired resistance had been proposed [9].

#### 2.3.1. Protoplast Secretion Studies

We aimed to shed light on AZI1 intercellular mobility. We also asked whether intracellular/cell-bound AZI1 molecules would differ in size from potentially secreted ones. Generally, proteins entering the secretory pathway may become entrapped in the cell wall and/or be released into the apoplastic fluid. Only in the latter case would such proteins be potentially mobile signals. We decided to conduct analyses on AZI1 secretion in a cell wall-free system. To this end, mesophyll protoplasts were isolated from *Arabidopsis* wild type and AZI1-myc overexpressing stable transgenic plants. Following thorough and gentle washing steps, protoplasts were resuspended in a small volume of protoplast incubation solution (W5/BSA). Protoplast suspensions from wild type and transgenic plants were visually indistinguishable, and they were virtually devoid of damaged cells or cell debris. The cells were allowed to settle by incubation of the suspension at 4 °C for two days. The reasoning was that during this period secretion-destined proteins should accumulate in the liquid surrounding the protoplasts. While keeping the settled protoplasts undisturbed, the incubation solution (“secretion fluid”) was removed. Light microscopy of the settled protoplasts documented that during the two days incubation, cells had remained intact. Importantly, the collected fluid was devoid of any protoplasts or cell debris, and centrifugation of this fluid yielded no visible pellet.

**Figure 4:**
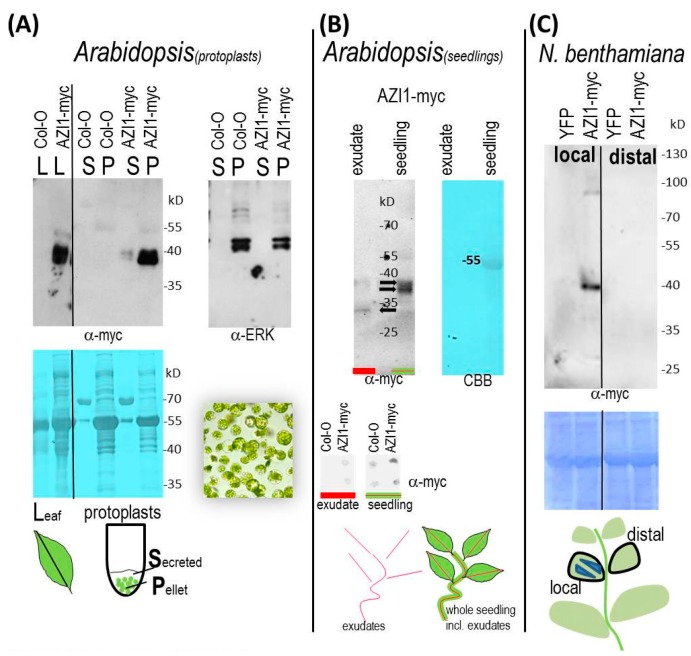
AZI1 secretion and mobility. (**A**) AZI1 secreted from protoplasts. Immunoblot detection of AZI1-myc protein in *Arabidopsis* leaves (L), extracts from settled mesophyll protoplasts (P) and secreted fluids collected after two days (S). Protoplasts were fully intact (light microscopy, bottom right image); and an antibody against cytosolic protein (a-ERK, right) yielded detectable signals only in protoplast extracts, not in secreted fluids. Protein loading was documented by Coomassie brilliant blue (CBB) staining of the PVDF membranes (**bottom**). Note that the abundant bands in secretion fluids (stained with CBB) were derived from BSA that is contained in the protoplast incubation medium; (**B**) Presence of AZI1 in *Arabidopsis* seedling exudates. Exudates and total protein extracts from 15-day-old seedlings of *AZI1*-myc transgenic lines were examined with anti-myc antibody. Immunoreactive bands observed in the immunoblot (**left**) are indicated by arrows. Absence of RuBisCo (Ru) from the exudates was documented by subsequent CBB-staining of the PVDF membrane (**right**). For dot blot analysis (**bottom**), exudates and seedling total protein extracts were derived from control Col-0 and *AZI1*-myc transgenic lines. Experiments were repeated twice; (**C**) Long-distance movement *in vivo:* *N. benthamiana* leaves were infiltrated with agrobacteria carrying the YFP (control) or AZI1-myc transgene. Upon subsequent immunoblot analysis with anti-myc antibody AZI1 was detectable in protein extracts from the infiltrated area (marked in blue, bottom image), but not in distal leaves. The relative position of leaves used in these experiments is indicated (border line, bottom image).

The presence of AZI1-myc proteins was subsequently assessed in the secretion fluid as well as in protoplast protein extracts. In samples from AZI1-myc, but not from control Col-0 plants, 36/38 kD-sized immunoreactive bands were observed with anti-myc antibody (Figure 4A). Transgene-specific signals were primarily seen in protein extracts from cells (P) but also in the incubation fluid (S). These findings indicate that a proportion of AZI1 is secreted by protoplasts. The signal that remains in the pellet might be derived from intracellular *i.e.*, ER-localized AZI1-myc *en route* to secretion. The much stronger signal intensity in the pellet fraction, as compared to that in the secreted fluid suggests that secretion is not the only destination. A substantial proportion of AZI1 likely remains entrapped in the cell, most likely at the plasma membrane via lipophilic interaction of the 8-CM domain. A membrane-association has been demonstrated for EARLI1-YFP fusion proteins [5] as well as for AZI1-MPK3 protein complexes *in vivo* [6].

No qualitative differences in gel migration properties between cellular and secreted AZI1 exist, implying that the yet-elusive posttranslational modification remains and that intra- and extra-cellular molecules are likely chemically identical.

#### 2.3.2. AZI1 Detection in Seedling Exudates

To gain confidence in AZI1 secretion, presence of the protein was examined in apoplastic fluids (Figure 4B). To this end, exudates were collected from *Arabidopsis* wild type and stable AZI1-myc transgenic seedlings. Immunodetection with anti-myc antibody was conducted by dot blot analysis as well as by immunoblotting following separation by SDS-PAGE. Seedlings were handled with extreme care to avoid tissue damage. Exudates and proteins extracted from seedling tissue (remaining after exudate collection) were examined in parallel. Dot blot and SDS-PAGE/immunoblot analyses of exudates yielded detectable signals exclusively in samples of AZI1-myc overexpressing plants. Subsequent Coomassie staining of membranes confirmed absence of any abundant protein in exudate samples (which would be an indicator of tissue damage). On dot blots (Figure 4B, bottom), weak immunoreactive signals were found in Col-0 wild type tissue extracts (remaining after exudate collection) plants, representing some non-specific binding of the antibody. In comparison, signals were more pronounced in the corresponding samples of AZI1-myc plants. Dot blot analyses may therefore be considered a suitable approach for studying stimulus-triggered changes in AZI1 secretion; an issue of our ongoing research.

When comparing SDS-PAGE-separated AZI1-myc-specific bands in exudates *vs.* seedling protein extracts a shift in size can be noted (Figure 4B, top). Seedlings contain the characteristic 36/38 kD double band. A very faint band at approximately 32 kD also exists. In exudates, this 32 kD band predominates, while the 36/38 kD signal is only faint. The 32 kD-sized protein *may* represent the preferred secreted form of AZI1. Alternatively, this molecule results from extracellular proteolytic cleavage of the secreted 36/38 kD AZI1 variant. Data obtained from protoplast experiments are in favor of the second explanation. These observations raise the intriguing question about the participation of phloem-located proteases in AZI1-related systemic responses.

#### 2.3.3. AZI1 Long-Distance Mobility *in Vivo*—Experimental Approaches and Limitations

Given the observations on AZI1 secretion, we next aimed to get insight into the protein’s mobility *in planta*. Constitutively AZI1-overexpressing transgenics are unsuitable for such experiments because the transgene product would be produced in all parts of the plant. Even with organ-specific promoters a certain “leakiness” cannot be excluded. A system is needed in which transgene expression is locally restricted. *N. benthamiana* lower leaves were infiltrated with an agrobacterial suspension carrying a YFP (control), a AZI1-YFP or a AZI1-myc construct. After five, eight and 12 days, infiltrated leaves as well as non-infiltrated leaves were analyzed. YFP-infiltrated leaf areas exhibited strong fluorescence, while adjacent areas or distal leaves of the same plants were devoid of YFP-derived fluorescence. This documents high transformation efficiency; and it also implies that free YFP does not notably “travel” through the plant. Transgene-derived fluorescent signals were observed in leaf areas infiltrated with AZI1-YFP, but not outside this infiltration zone or in distal leaves (not shown). One possible limitation is the known attenuation of YFP fluorescence at low pH, as is found in the apoplast [44]. As an alternative, AZI1 was therefore expressed as myc-tagged fusion protein and assessed by immunoblotting (Figure 4C). Similar to the observations made with YFP-tagged AZI1, detectable AZI1-myc levels were confined to the infiltrated leaf areas. Compared to other transgenes (same promoter) we had worked with, the amount of AZI1 produced in the infiltrated area is low. The dilution effect if it moved into distal parts of the plant is a limiting point. The here-described approach discloses some limitations and forms the basis for optimization. Higher expression levels and less pH-sensitive fluorescence tags such as citrine YFP [45] shall provide a clearer answer on AZI1 long-distance mobility *in planta.*

## 3. Material and Methods

### 3.1. DNA Constructs and (de)-Phosphomimetic Arabidopsis Transgenic Lines

Accession numbers: AZI1 At4g12470, MPK3 At3g45640. The CaMV35S::AZI1-myc transgene for constitutive overexpression of C-terminally myc-tagged AZI1 was described earlier [6]. Putative MAPK phosphorylation sites (S33, S41, S59, T66, T70) were replaced by site-directed mutagenesis and cloned in *E. coli*. After verification by DNA sequencing, plasmids were transformed into *A. tumefaciens* (GV3101) harbouring the helper plasmid pSoup [46]. The floral dip method [47] was used to generate stable transgenic *Arabidopsis* lines CaMV35S::AZI1-AAA and CaMV35S::AZI1-DDDEE. Kanamycin resistant candidates were selected for seed propagation to yield homozygous lines. Transgene expression was assessed by immunoblot analysis with anti-myc antibody. Germination assays on ½ MS medium with/without 150 mM NaCl were conducted as previously described [6].

### 3.2. Plant Growth and Treatment

*Arabidopsis* Col-0 seeds were surface-sterilized and sown on solid standard medium (1/2 MS, 0.25% sucrose, 1% agar). After vernalization (4 °C, 3d, dark), plates were placed into an incubator (22 °C, 16/8h day/night regime). Twelve-day-old *Arabidopsis* seedlings were transferred to 12-well-plates containing 1 mL liquid standard medium (1 mL, 5 seedlings per well) and adapted overnight. Stresses were applied by adding 1 volume of 2 µM flg22 or 500 mM NaCl. For wounding, seedlings were punched with serrated forceps. To study prolyl hydroxylation, seedlings were incubated (4, 8, 24 h) in standard medium (1/2 MS) with or without 100 µM 2,2-dipyridyl (Sigma, St. Louis, MO, USA). Plants were shock-frozen and processed for protein extraction and immunoblot analyses.

Transient *Agrobacterium*-mediated transformation of *N. benthamiana* and *T. majus* was performed as previously described [29,30]. For long-distance movement studies of AZI1, independent, similar-sized *N. benthamiana* plants (nine-leaf-stage) were used for each AZI1 derivative. Material from the infiltrated leaf (“local”), the adjacent area as well as from the immediately following upper leaf (“distal”) was collected for immunoblot analysis.

### 3.3. Isolation of Protoplasts and Secreted Fluid

Mesophyll protoplasts were isolated from similar-sized, fully-expanded, healthy leaves of Col-0 wild type and AZI1-overexpressing *Arabidopsis* plants using a modified version [48] of the “tape-*Arabidopsis*-sandwich” protocol [49]. Protoplasts were washed in W5/BSA medium. Protoplast suspensions (approximately 50% cells, 50% medium) were incubated in vertical tubes at 4 °C without agitation for two days. Cells had completely settled within this period. Cell integrity and absence of cell debris was documented by light microscopy, before and after the incubation period. The supernatant fluid devoid of cells or cell debris, and the settled intact protoplasts were snap-frozen and assessed in parallel by immunoblot analysis with anti-myc antibody.

### 3.4. Isolation of Apoplastic Fluid from Arabidopsis Thaliana Seedlings

Apoplastic fluids (exudates) were collected from 20–30, 15-day-old seedlings. To this end, seedlings were placed in a mesh, which was submerged in a prechilled container containing ice-cold buffer solution (100 mM Tris–HCl pH 7.5, 100 mM MgCl_2_, 2 mM EDTA). Small weights were used to keep the mesh with seedlings submerged in the buffer throughout the infiltration procedure. Vacuum was applied to infiltrate the buffer for 8–10 min (till bubbles appeared on the surface of the mesh) and then released slowly. Infiltrated seedlings looked translucent. After infiltration, the seedlings were dry-blotted gently with a paper towel and placed in a new mesh. This mesh was tightened in the windings of a 50 mL conical plastic tube and centrifuged for 15 min at 1000× *g* at 4 °C. The apoplastic fluids (approximately 20 µL obtained from 30 seedlings) were then used for SDS-PAGE/Western blot or dot blot analysis.

### 3.5. Protein Extraction and Immunoblot Analysis

Proteins were extracted from snap-frozen *Arabidopsis* (seedlings, leaves and protoplasts), *N. benthamiana* (leaves) and *T. majus* (leaves). Frozen tissue was ground to a fine powder using liquid nitrogen and a homogenizer mill. Two volumes of extraction buffer (50 mM Tris–HCl, pH 7.5, 5 mM EDTA pH 8, 5 mM EGTA pH 8, 2 mM DTT, 100 mM β-glycerophosphate, 10 M Na–Vanadat, 10 mM Na–Fluorid, 10 mM PMSF, 10 µg/mL aprotinin, 10 µg/mL leupeptin) were added, and samples were incubated on ice for 10 min. Protein concentration in the supernatant liquids obtained after 15 min centrifugation (4 °C, 14,000× *g*) were adjusted to 3 µg/µL (quantified by Bradford assay, using BSA as standard) by adding the respective volume of extraction buffer. Fifty µL protein extracts, supplemented with 10 µL 6× protein Laemmli loading dye (incl. DTT), were denatured at 95 °C for 5 min. For dot blots, non-denatured 2 µL (exudate) or 3 µg protein (seedling extract) were used. Proteins were separated by SDS-PAGE and blotted onto PVDF membranes. Membranes were incubated with primary antibodies (1:3000) rabbit anti-myc (NEB signaling) at 4 °C over night, washed in TBST (3 × 5 min) and subsequently incubated with secondary antibody (1:20,000) anti-rabbit800 CW (NEB signaling) for 1 h at room temperature. Bound infrared dye-coupled secondary antibodies were detected by scanning the washed membranes (TBST, 3 × 5 min) with an Odyssey imaging system. Protein loading was documented by subsequent staining of membranes with Coomassie Blue. Immunodetection of glycosylation was performed with primary antibodies (MYC207, LM2 and LM14) and horse-radish-peroxidase-coupled secondary antibodies. Chemiluminescent detection of HRP-catalysed luminol oxidation was performed using a ChemiDoc imager system.

### 3.6. Immunoprecipitation

For immunoprecipitation, protein extracts were prepared from 3-week-old *Arabidopsis* seedlings (Col-0 as control and AZI1-myc overexpressing line) as described above, using binding buffer B+ (100 mM Tris–HCl, pH 7.5, 75 mM NaCl, 1 mM EDTA, 0.1% triton, 0.05% SDS, 10% glycerol, 2 mM DTT, 10 mM Na–Vanadate, 10 mM Na–Fluoride, 10 mM PMSF, 10 µg/mL aprotinin, 10 µg/mL leupeptin). Three hundred µL protein extracts, adjusted to 3 µg/µL in B+, were incubated with 2 µL of mouse anti-c-myc antibody (C10) on a rotating wheel at 4 °C over night. Samples were briefly centrifuged, liquids were transferred into two fresh tubes containing 20 µL proteinA-coupled magnetic beads (Dynabeads) which had been equilibrated in extraction buffer B− (lacking protease inhibitors). The mixtures were returned to the rotating wheel at 4 °C for 3 h. Beads containing bound proteins were collected using a magnetic rack and washed 1× in 500 µL and 2× in 200 µL buffer B−. To avoid possible elution of (non-specific) proteins that may have bound to the sample tube surface, beads were resuspended in 50 µL buffer A and transferred into fresh tubes. The liquid was removed, and proteins were finally eluted in 30 µL 1× Laemmli loading dye (including DTT) at 95 °C for 5 min. Samples were processed for immunoblot analysis as described above.

## 4. Conclusions

At present the available evidence for post-translational modification is the reduction-insensitive but Prolyl-4-hydroxylase-sensitive discrepancy between apparent molecular weight compared to the unmodified protein. Since there are no *N*-glycosylation sites but several potential *O*-glycosylation sites (PSP, TPP; see [19]) this posttranslational modification is the most likely explanation of the discrepancy.

Given the consistent size pattern of AZI1-myc and AZI1-quintuple mutant derivatives one can conclude that AZI1 putative *O*-glycosylation and MAPK-targeted phosphorylation sites do not overlap, and that *O*-glycosylation unlikely occurs at residues Ser33, Ser41, Ser59, Thr66 or Thr70. Our data point to AZI1 being a hydroxyproline-rich glycoprotein. This posttranslational modification is seemingly constitutive, because AZI1 retarded gel migration occurred in an organ-, developmental stage- and treatment-independent manner. AZI1 gel migration was retarded, irrespective of whether the transgene was expressed in *Arabidopsis*, *N. benthamiana* or *T. majus*. This is not unusual; similarity in polysaccharide structure between widely separated plant families had been noted earlier [50]. Nonetheless, conclusive evidence for glycosylation of AZI1 is still lacking. A Pro-rich stretch of (ProXaa)_8_ and (ProXaa)_4_ in the N-terminus of AZI1 has strong similarity to AGPs, though it had not been classified as such before (Supplemental table 5 in [51]). A clear answer may be obtained by MALDI-TOF and HPLC-based approaches that require substantial amounts of plant material (reviewed e.g., in [21,27,31]). Neither AZI1 nor any other pEARLI-like proteins are on the list of *Arabidopsis* HRGPs identified by a bioinformatics-based analysis [19]. Although several families such as extensins and AGPs have been classified into HRGP subgroups due to highly repetitive Pro-rich repeats, some groups such as fasciclin-like AGPs and chimeric AGPs were identified due to previous proteomic or biochemical studies of individual, typically highly expressed or biologically active, group members [21,52,53,54]. The recently identified tracheary element differentiation factor xylogen from zinnia is a GPI-anchored AGP with similarities to non-specific LTPs [52]. Its LTP region is 35% identical and 59% similar to AZI1 and its short N-terminal Pro-rich region has been determined to contain non-contiguous Hyp residues. This observation and our identification of AZI1 as likely HRGP, suggests that the size of this highly diverse protein family is still underestimated. Our data from *in vivo* experiments point to AZI1 being a partially secreted protein, a feature unknown for any MAPK substrate so far. Its dual location suggests AZI1 to exert both intra- and extracellular functions. The protein clearly deserves further study, for which we hope to provide inspiration. Future work on AZI1 mutated variants may include an investigation of whether AZI1-conferred stress resistance is primarily due to the cellular or extracellular pool of the protein, and whether glycosylation is essential for protein function. Data arising from such studies have a potentially high impact, given the evolutionary conservation of MAPK-mediated stress signaling, the recognition of AZI1 by heterologous post-translational machineries and the wide distribution of close AZI1 homologs in the plant kingdom.

## References

[1] Dvorakova L., Cvrckova F., Fischer L. (2007). Analysis of the hybrid proline-rich protein families from seven plant species suggests rapid diversification of their sequences and expression patterns. BMC Genom..

[2] Jose-Estanyol M., Gomis-Ruth F.X., Puigdomenech P. (2004). The eight-cysteine motif, a versatile structure in plant proteins. Plant Physiol. Biochem..

[3] Silverstein K.A., Moskal W.A., Wu H.C., Underwood B.A., Graham M.A., Town C.D., VandenBosch K.A. (2007). Small cysteine-rich peptides resembling antimicrobial peptides have been under-predicted in plants. Plant J..

[4] Richards K.D., Schott E.J., Sharma Y.K., Davis K.R., Gardner R.C. (1998). Aluminum induces oxidative stress genes in arabidopsis thaliana. Plant Physiol..

[5] Zhang Y., Schlappi M. (2007). Cold responsive earli1 type hyprps improve freezing survival of yeast cells and form higher order complexes in plants. Planta.

[6] Pitzschke A., Datta S., Persak H. (2014). Salt stress in arabidopsis: Lipid transfer protein azi1 and its control by mitogen-activated protein kinase mpk3. Mol. Plant.

[7] Shi Y., Zhang X., Xu Z.Y., Li L., Zhang C., Schlappi M., Xu Z.Q. (2011). Influence of earli1-like genes on flowering time and lignin synthesis of arabidopsis thaliana. Plant Biol..

[8] Xu D., Huang X., Xu Z.Q., Schlappi M. (2011). The hyprp gene earli1 has an auxiliary role for germinability and early seedling development under low temperature and salt stress conditions in arabidopsis thaliana. Planta.

[9] Jung H.W., Tschaplinski T.J., Wang L., Glazebrook J., Greenberg J.T. (2009). Priming in systemic plant immunity. Science.

[10] Atkinson N.J., Lilley C.J., Urwin P.E. (2013). Identification of genes involved in the response of arabidopsis to simultaneous biotic and abiotic stresses. Plant Physiol..

[11] Yu K., Soares J.M., Mandal M.K., Wang C., Chanda B., Gifford A.N., Fowler J.S., Navarre D., Kachroo A., Kachroo P. (2013). A feedback regulatory loop between g3p and lipid transfer proteins dir1 and azi1 mediates azelaic-acid-induced systemic immunity. Cell Rep..

[12] Andreasson E., Ellis B. (2010). Convergence and specificity in the arabidopsis mapk nexus. Trends Plant Sci..

[13] Colcombet J., Hirt H. (2008). Arabidopsis mapks: A complex signalling network involved in multiple biological processes. Biochem. J..

[14] Pitzschke A., Schikora A., Hirt H. (2009). Mapk cascade signalling networks in plant defence. Curr. Opin. Plant Biol..

[15] Popescu S.C., Popescu G.V., Bachan S., Zhang Z., Gerstein M., Snyder M., Dinesh-Kumar S.P. (2009). Mapk target networks in arabidopsis thaliana revealed using functional protein microarrays. Genes Dev..

[16] Pitzschke A. (2015). Modes of mapk substrate recognition and control. Trends Plant Sci..

[17] Pitzschke A., Datta S., Persak H. (2014). Mitogen-activated protein kinase-regulated azi1—An attractive candidate for genetic engineering. Plant Signal. Behav..

[18] Beckers G.J., Jaskiewicz M., Liu Y., Underwood W.R., He S.Y., Zhang S., Conrath U. (2009). Mitogen-activated protein kinases 3 and 6 are required for full priming of stress responses in arabidopsis thaliana. Plant Cell.

[19] Showalter A.M., Keppler B., Lichtenberg J., Gu D., Welch L.R. (2010). A bioinformatics approach to the identification, classification, and analysis of hydroxyproline-rich glycoproteins. Plant Physiol..

[20] Showalter A.M. (1993). Structure and function of plant-cell wall proteins. Plant Cell.

[21] Schultz C.J., Johnson K.L., Currie G., Bacic A. (2000). The classical arabinogalactan protein gene family of arabidopsis. Plant Cell.

[22] Ellis M., Egelund J., Schultz C.J., Bacic A. (2010). Arabinogalactan-proteins: Key regulators at the cell surface?. Plant Physiol..

[23] Showalter A.M. (2001). Arabinogalactan-proteins: Structure, expression and function. Cell. Mol. Life Sci..

[24] Kieliszewski M.J., Lamport D.T. (1994). Extensin: Repetitive motifs, functional sites, post-translational codes, and phylogeny. Plant J..

[25] Tan L., Qiu F., Lamport D.T., Kieliszewski M.J. (2004). Structure of a hydroxyproline (hyp)-arabinogalactan polysaccharide from repetitive ala-hyp expressed in transgenic *nicotiana tabacum*. J. Biol. Chem..

[26] Tryfona T., Liang H.C., Kotake T., Tsumuraya Y., Stephens E., Dupree P. (2012). Structural characterization of arabidopsis leaf arabinogalactan polysaccharides. Plant Physiol..

[27] Hijazi M., Durand J., Pichereaux C., Pont F., Jamet E., Albenne C. (2012). Characterization of the arabinogalactan protein 31 (AGP31) of arabidopsis thaliana: New advances on the hyp-o-glycosylation of the pro-rich domain. J. Biol. Chem..

[28] Wang H., Ngwenyama N., Liu Y., Walker J.C., Zhang S. (2007). Stomatal development and patterning are regulated by environmentally responsive mitogen-activated protein kinases in arabidopsis. Plant Cell.

[29] Persak H., Pitzschke A. (2013). Tight interconnection and multi-level control of arabidopsis myb44 in mapk cascade signalling. PLoS ONE.

[30] Pitzschke A. (2013). Tropaeolum tops tobacco - simple and efficient transgene expression in the order brassicales. PLoS ONE.

[31] Wilson I.B. (2002). Glycosylation of proteins in plants and invertebrates. Curr. Opin. Struct. Biol..

[32] Kieliszewski M.J. (2001). The latest hype on hyp-o-glycosylation codes. Phytochemistry.

[33] Bardor M., Faye L., Lerouge P. (1999). Analysis of the *N*-glycosylation of recombinant glycoproteins produced in transgenic plants. Trends Plant Sci..

[34] Tryfona T., Liang H.C., Kotake T., Kaneko S., Marsh J., Ichinose H., Lovegrove A., Tsumuraya Y., Shewry P.R., Stephens E. (2010). Carbohydrate structural analysis of wheat flour arabinogalactan protein. Carbohyd. Res..

[35] Pattathil S., Avci U., Baldwin D., Swennes A.G., McGill J.A., Popper Z., Bootten T., Albert A., Davis R.H., Chennareddy C. (2010). A comprehensive toolkit of plant cell wall glycan-directed monoclonal antibodies. Plant Physiol..

[36] Knox J.P. (1997). The use of antibodies to study the architecture and developmental regulation of plant cell walls. Int. Rev. Cytol..

[37] Nguema-Ona E., Coimbra S., Vicre-Gibouin M., Mollet J.C., Driouich A. (2012). Arabinogalactan proteins in root and pollen-tube cells: Distribution and functional aspects. Ann. Bot..

[38] Knox J.P., Linstead P.J., Peart J., Cooper C., Roberts K. (1991). Developmentally regulated epitopes of cell-surface arabinogalactan proteins and their relation to root-tissue pattern-formation. Plant J..

[39] Moller I., Marcus S.E., Haeger A., Verhertbruggen Y., Verhoef R., Schols H., Ulvskov P., Mikkelsen J.D., Knox J.P., Willats W. (2008). High-throughput screening of monoclonal antibodies against plant cell wall glycans by hierarchical clustering of their carbohydrate microarray binding profiles. Glycoconj. J..

[40] Speake B.K., Hemming F.W., White D.A. (1980). The effects of tunicamycin on protein glycosylation in mammalian and fungal systems. Biochem. Soc. Trans..

[41] Barnett N.M. (1970). Dipyridyl-induced cell elongation and inhibition of cell wall hydroxyproline biosynthesis. Plant Physiol..

[42] Fernandez-Calvino L., Faulkner C., Walshaw J., Saalbach G., Bayer E., Benitez-Alfonso Y., Maule A. (2011). Arabidopsis plasmodesmal proteome. PLoS ONE.

[43] Mitra S.K., Walters B.T., Clouse S.D., Goshe M.B. (2009). An efficient organic solvent based extraction method for the proteomic analysis of arabidopsis plasma membranes. J. Proteom. Res..

[44] Young B., Wightman R., Blanvillain R., Purcel S.B., Gallois P. (2010). pH-sensitivity of YFP provides an intracellular indicator of programmed cell death. Plant Method.

[45] Griesbeck O., Baird G.S., Campbell R.E., Zacharias D.A., Tsien R.Y. (2001). Reducing the environmental sensitivity of yellow fluorescent protein. Mechanism and applications. J. Biol. Chem..

[46] Hellens R.P., Edwards E.A., Leyland N.R., Bean S., Mullineaux P.M. (2000). Pgreen: A versatile and flexible binary ti vector for agrobacterium-mediated plant transformation. Plant Mol. Biol..

[47] Clough S.J., Bent A.F. (1998). Floral dip: A simplified method for agrobacterium-mediated transformation of arabidopsis thaliana. Plant J..

[48] Pitzschke A., Persak H. (2012). Poinsettia protoplasts—A simple, robust and efficient system for transient gene expression studies. Plant Methods.

[49] Wu F.H., Shen S.C., Lee L.Y., Lee S.H., Chan M.T., Lin C.S. (2009). Tape-arabidopsis sandwich—A simpler arabidopsis protoplast isolation method. Plant Methods.

[50] Shpak E., Leykam J.F., Kieliszewski M.J. (1999). Synthetic genes for glycoprotein design and the elucidation of hydroxyproline-o-glycosylation codes. Proc. Natl. Acad. Sci. USA.

[51] Ma H., Zhao J. (2010). Genome-wide identification, classification, and expression analysis of the arabinogalactan protein gene family in rice (*Oryza sativa* L.). J. Exp. Bot..

[52] Motose H., Sugiyama M., Fukuda H. (2004). A proteoglycan mediates inductive interaction during plant vascular development. Nature.

[53] Baldwin T.C., Domingo C., Schindler T., Seetharaman G., Stacey N., Roberts K. (2001). Dcagp1, a secreted arabinogalactan protein, is related to a family of basic proline-rich proteins. Plant Mol. Biol..

[54] Johnson K.L., Jones B.J., Bacic A., Schultz C.J. (2003). The fasciclin-like arabinogalactan proteins of arabidopsis. A multigene family of putative cell adhesion molecules. Plant Physiol..

